# Discordance gradient-surface dans le rétrécissement mitral: le gradient moyen transmitral est-il un critère de sévérité ou un indice de tolérance du rétrécissement mitral serré?

**DOI:** 10.11604/pamj.2016.25.75.8797

**Published:** 2016-10-06

**Authors:** Hayat Najih, Salim Arous, Aziza Laarje, Dalila Baghdadi, Mohamed Ghali Benouna, Leila Azzouzi, Rachida Habbal

**Affiliations:** 1 Service de Cardiologie, CHU Ibn Rochd, Casablanca, Maroc

**Keywords:** Rétrécissement mitral serré, gradient moyen transmitral, surface valvulaire mitrale, Severe mitral stenosis, mean transmitral gradient, surface of the mitral valve

## Abstract

Le rétrécissement mitral (RM) rhumatismal demeure une valvulopathie fréquente dans les pays en voie de développement. Cependant, les pays industrialisés ont vu l'émergence ces dernières années de nouvelles étiologies de RM; notamment l'origine médicamenteuse et/ou toxique responsable de valvulopathies restrictives aussi bien sténosantes que régurgitantes. Pour cette raison, l'évaluation échocardiographique du RM et surtout, la définition de critères objectifs pour conclure au caractère serré du RM reste toujours d'actualité. Les objectifs du travail sont: évaluer l'existence ou non d'une corrélation directe entre le gradient moyen transmitral (GMT) et la sévérité du RM chez les patients porteurs d'un RM serré ou très serré (critère primaire) et analyser les différents paramètres qui conditionnent le gradient moyen transmitral (GMT) (Critère secondaire). Il s'agit d'une étude transversale monocentrique incluant tous les patients admis au service de Cardiologie du CHU Ibn Rochd de Casablanca pour un RM serré ou très serré, sur une période d'une année (Janvier 2014 à Décembre 2014). Nous avons analysés séparément deux groupes de patients : ceux avec un gradient moyen transmitral<10 mmHg (groupe 1) et ceux avec un gradient>10mmHg (groupe2). 50 patients porteurs d'un RM serré ou très serré ont été inclus. L'âge moyen de nos patients est de 41,7 ans avec prédominance féminine (sex ratio: 0,25). 64% de nos patients avaient un RM serré et 36% avaient un RM très serré. 52% (26 patients) avaient un GMT <10mmHg et 48% (24 patients) avaient un gradient moyen >10mmHg, ce qui suggère l'absence de corrélation directe entre la sévérité du RM et le GMT (coefficient de Pearson R: -0,137). Pour la dyspnée, 80% des patients du groupe 1 étaient dyspnéiques stade II de la NYHA et 70% des patients du groupe 2 étaient dyspnéiques stade III (41%) ou IV (29%) de la NYHA, ce qui signifie l'existence d'une corrélation significative entre le GMT et la sévérité de la dyspnée (R: 0,586 et p: 0,001). L'étude analytique de la fréquence cardiaque et la présence d'une décompensation cardiaque par rapport au gradient moyen transmitral a montré une corrélation significative. En effet, parmi les patients du groupe 1, 96% avaient une FC entre 60 et 100 bpm et aucun patient n'était en décompensation cardiaque. Dans le groupe 2,54% (13 patients) avaient une FC >100 bpm et 7 parmi eux (53%) étaient en décompensation cardiaque gauche. L'étude de la pression artérielle pulmonaire systolique dans les deux groupes de l'étude a révélé l'existence dans notre série d'une corrélation statistiquement significative (R : 0,518 et P : 0,001) entre la PAPS et le GMT. La régularité du rythme et la fonction ventriculaire droite n'étaient pas corrélées au GMT (R : 0,038 et R :-0,002 respectivement). Le gradient moyen transmitral est un bon indicateur de la tolérance du rétrécissement mitral, mais il reflète imparfaitement sa sévérité vue qu'il dépend de plusieurs paramètres hémodynamiques. D'authentiques rétrécissements mitraux très serrés peuvent exister avec des gradients moyens transmitraux <10 mm Hg, c'est pour cette raison que la valeur du GMT ne doit jamais être interprétée isolément.

## Abstract (en)

Rheumatic mitral valve stenosis (MVS) is a frequent valvulopathy in developing countries. However, industrialized countries have seen the emergence of new etiologies of MVS in recent years, in particular drug-induced and/or toxic valvular regurgitation and stenosis. For this reason, the echocardiographic assessment of MVS and especially the definition of objective diagnostic criteria for severe MVS remains relevant. The objectives are: to determine whether there is a direct causal link between mean transmitral gradient (MTG) and severity of MVS in patients with severe MVS or true severe MVS (primary criterion); to analyze different parameters determining mean transmitral gradient (MTG) (secondary criterion). We conducted a single-center cross-sectional study including all patients with severe or true severe MVS admitted to the Department of Cardiology, University Hospital Ibn Rushd, Casablanca over a period of one year (January 2014-December 2014). We analyzed data from two groups of patients separately: those with a mean transmitral gradient<10 mmHg (group 1) and those with a gradient>10mmHg (group 2). 50 patients with severe or true severe MVS have been included in the study. The average age of our patients was 41.7 years with a female predominance (sex ratio 0,25). 64% of patients had severe MVS and 36% of patients had true severe MVS. 52% (26 patients) had MTG < 10mmHg and 48% (24 patients) had mean gradient> 10mmHg, suggesting no direct correlation between the severity of MVS and MTG (Pearson's correlation coefficient R: -0,137). With regards to dyspnea, 80% of patients of group 1 had stage II NYHA dyspnea (classification system) and 70% of patients of group 2 had stage III NYHA dyspnea (41%) or IV NYHA dyspnea (29%), which means that there was a significant correlation between MTG and the severity of dyspnea (R: 0,586 and p: 0,001). The analytical study of heart rate and the presence of cardiac decompensation compared with mean gradient transmitral showed a significant correlation. Indeed, among patients in group 1, 96% had HR between 60 and 100 bpm and no patient had decompensated heart failure. In group 2, 54% (13 patients) had a HR> 100 bpm and 7 of them (53%) had left decompensated heart failure. The analysis of systolic pulmonary artery pressure conducted in both groups of the study revealed the existence of a statistically significant correlation (R: 0,518 and P: 0,001) between systolic pulmonary artery pressure (SPAP) and MTG. Ventricular rhythm regularity and right ventricular function were not correlated with MTG (R: 0,038 and R: - 0,002 respectively). Mean transmitral gradient is a good indicator of mitral stenosis tolerance but it imperfectly reflects mitral stenosis severity as this depends on several hemodynamic parameters. True severe mitral stenosis may have mean transmitral gradient < 10mmHg, that is why the value of MTG should never be interpreted as single value.

## Introduction

Le rétrécissement mitral (RM), fréquent autrefois, est devenu rare dans les pays occidentaux, du fait de la quasi-disparition du rhumatisme articulaire aigu (RAA). La situation est tout autre dans les pays en voie de développement, où le RAA et les valvulopathies rhumatismales restent répandus, et les formes serrées de RM peuvent exister dès le jeune âge. Cependant, les pays industrialisés ont vu l'émergence ces dernières années de nouvelles étiologies de RM ; notamment l'origine médicamenteuse et/ou toxique responsable de valvulopathies restrictives aussi bien sténosantes que régurgitantes. Pour toutes ces raisons, l'évaluation échocardiographique du RM et surtout, la définition de critères objectifs pour conclure au caractère serré du RM reste toujours d'actualité. Les sociétés savantes internationales définissent le RM serré par une surface valvulaire mitrale = à 1,5 cm^²^ et un RM très serré par une surface valvulaire mitrale 1cm^²^ avec un gradient moyen transmitral >10 mmHg [1]. Toutefois, malgré l'existence de discordances concernant la valeur cut-off du gradient moyen transmitral (GMT) qui permet de définir la sévérité du RM et la variation de ce gradient en fonction de plusieurs facteurs (fréquence cardiaque, régularité du rythme, valvulopathies associées, débit cardiaque), le gradient moyen transmitral figure encore dans les recommandations comme critère de sévérité du RM. Les objectifs de notre travail est d': évaluer l'existence ou non d'une corrélation directe entre le gradient moyen transmitral et la sévérité du RM chez les patients porteurs d'un RM serré ou très serré (critère primaire); analyser les différents paramètres qui conditionnent le gradient moyen transmitral. (critère secondaire).

## Méthodes

Nous avons réalisé une étude transversale monocentrique sur une année (janvier 2014 à décembre 2014) incluant tous les patients admis au service de Cardiologie du CHU Ibn Rochd de Casablanca pour RM serré ou très serré. Ont été exclu de l'étude : tous les patients porteurs d'un RM moyennement serré ou peu serré; les patients porteurs d'une valvulopathie mitrale et/ou aortique significative associée au RM; les autres cardiopathies congénitales ou acquises associées au RM.

Tous nos patients ont bénéficié de : un interrogatoire détaillé révélant leurs données démographiques, leurs symptômes, leurs facteurs de risque cardiovasculaire et leurs antécédents, notamment l'existence d'un accident embolique; un examen clinique complet; un électrocardiogramme (ECG) 12 dérivations ; une échocardiographie Transthoracique (ETT) faite par un échographiste expérimenté, sur un appareil d'échocardiographie GE VIVID7. Ont été analysé en ETT la qualité de l'appareil valvulaire et sous valvulaire mitral, la surface valvulaire mitrale par planimétrie, le gradient moyen transmitral, la surface des oreillettes gauche et droite par planimétrie, la fraction d'éjection ventriculaire gauche en Teicholtz et en Simpson biplan, la fonction ventriculaire droite, l'insuffisance tricuspide (IT), le gradient VD-OD à partir du flux de l'IT et les POD à partir du diamètre et de la collapsibilité de la veine cave inférieure. La surface valvulaire mitrale retenue était calculée par méthode de planimétrie, en moyennant au moins 3 mesures.

Le gradient moyen transmitral (GMT) était calculé par le contournage du flux antérograde mitral obtenu par doppler continu sur l'orifice mitral, en moyennant au moins 3 mesures en rythme sinusal et 5 mesures en cas de fibrillation atriale. Dans un premier temps, nous avons étudié la corrélation entre la surface valvulaire mitrale et le GMT. Ensuite, nous avons analysés séparément deux groupes de patients : ceux avec un gradient moyen transmitral <10 mmHg (groupe 1) et ceux avec un gradient >10mmHg (groupe2). Nous avons réalisé pour chaque groupe une corrélation uni-variée du GMT avec le stade de la dyspnée selon la classification NYHA, la fréquence cardiaque, la décompensation cardiaque, la régularité du rythme, la surface de l'oreillette gauche calculée par planimétrie, la fonction VD, l'importance de l'insuffisance tricuspide et la pression artérielle pulmonaire systolique (PAPS). Les données descriptives et analytiques ont été obtenues par le logiciel SPSS version 20.

## Résultats

Cinquante patients porteurs d'un RM serré ou très serré ont été colligés au service de cardiologie du CHU Ibn Rochd de Casablanca sur une période d'une année (Janvier 2014 à Décembre 2014). L'âge moyen de nos patients est de 41,7 ans avec des extrêmes allant de 22 ans à 66 ans et une prédominance féminine (sex ratio : 0,25). 30 patients (60%) étaient âgés de <45 ans, 19 patients (38%) avaient un âge compris entre 45 et 65 ans et 1 patient (2%) avait plus de 65 ans. Un facteur de risque cardiovasculaire a été retrouvé chez 36% des patients. 4 patients (8%) étaient tabagiques, 2 diabétiques (4%), 5 hypertendus (10%) et 7 femmes ménopausées (14%). Un accident embolique à type d'AVCI a été retrouvé chez 20% de nos malades (soit 10 patients). Dans notre série, 2 patientes étaient enceintes au deuxième trimestre. Une patiente était dyspnéique stade II de la NYHA et l'autre dyspnéique stade III de la NYHA. 64% de nos patients avaient un RM serré et 36% avaient un RM très serré (Tableau 1). Parmi l'ensemble de nos patients, 52% (26 patients) avaient un GMT <10mmHg et 48% avaient un gradient moyen >10mmHg. Ceci suggère l'absence de corrélation directe entre la sévérité du RM et le GMT vue la présence d'authentiques RM serrés ou très serrés avec des GMT inférieurs à 10 mm Hg (coefficient de Pearson R: -0,137) (Tableau 2).

**Tableau 1:** Caractéristiques démographiques et cliniques de la population étudiée

CARACERISTIQUES	
**Nombre total de patients**	**50**
**Age moyen (années)**	41,7
<45 ans	30 patients (60%)
Entre 45 et 65 ans	19 patients (38%)
>65 ans	1 patient (2%)
**Sex ratio (male/ femmes)**	0,25
**Facteurs de risque cardiovasculaires**	18 patients (36%)
Tabac	4 patients (8%)
Diabète	2 patients (4%)
HTA	5 patients (10%)
Ménopause	7 patients (14%)
Antécédents d’AVCI	10 patients (20%)
**Fréquence cardiaque**	
<60 bpm	1 patient (2%)
Entre 60 et 100 bpm	36 patients (72%)
>100 bpm	13 patients (26%)
**Dyspnée (stade NYHA)**	
Stade I de la NYHA	1 patient (2%)
Stade II de la NYHA	28 patients (56%)
Stade III de la NYHA	14 patients (28%)
Stade IV de la NYHA	7 patients (14%)
**Durée d’évolution des symptômes**	
< 2 ans	31 patients (62%)
> 2ans	19 patients (38%)
**Décompensation cardiaque**	7 patients (14%)
**ECG**	
Rythme régulier sinusal (RRS)	26 patients (52%)
Fibrillation atriale	24 patients (48%)
RM serré <1,5 cm²	32 patients (64%)
RM très serré <1 cm²	18 patients (36%)

**Tableau 2:** Corrélation entre la surface mitrale et le GMT

	SM <1,5 cm²	SM<1 cm²	Total de patients	Coefficient de Pearson
GROUPE 1: Gradient moyen <10 mmHg	15	11	26	-0,137
GROUPE 2: Gradient moyen >10 mmHg	17	7	24	

Pour la dyspnée,80% des patients du groupe 1 étaient dyspnéiques stade II de la NYHA, 70% des patients du groupe 2 étaient dyspnéiques stade III (41%) ou IV (29%) de la NYHA et 80% des patients de notre série qui étaient dyspnéiques stade III ou IV de la NYHA avaient un GMT >10 mmHg (groupe 2), ce qui signifie l'existence d'une corrélation significative entre le GMT et la sévérité de la dyspnée (R: 0,586 et p: 0,001). L'étude analytique de la fréquence cardiaque et la présence d'une décompensation cardiaque par rapport au gradient moyen transmitral a montré une corrélation significative. En effet, parmi les patients du groupe 1, 96% avaient une FC entre 60 et 100 bpm et aucun patient n'était en décompensation cardiaque. Dans le groupe 2, 54% (13 patients) avaient une FC >100 bpm et 7 parmi eux (53%) étaient en décompensation cardiaque gauche.

En ce qui concerne la régularité du rythme, 53% des patients du groupe 1 (14 patients) étaient en Rythme régulier sinusal (RRS) et 46% (12 patients) étaient en fibrillation atriale. Dans le groupe 2, la moitié des patients était en RRS et la moitié en fibrillation atriale, ce qui signifie l'absence de corrélation statistiquement significative entre la régularité du rythme et le GMT dans notre série (R : 0,038). L'étude de la pression artérielle pulmonaire systolique dans les deux groupes de l'étude a révélé l'existence dans notre série d'une corrélation statistiquement significative (R: 0,518 et P: 0,001) entre la PAPS et le GMT. En effet, dans le groupe 1, 65% des patients (17 patients) avaient une PAPS normale <35 mmHg, 23% (6 patients) avaient une hypertension pulmonaire modérée avec une PAPS entre 35 et 50 mmHg et seulement 11% (3 patients) avaient une PAPS >50 mmHg. Dans le groupe 2, seulement 12% (3 patients) avaient une PAPS normale <35mmHg, 45% (11 patients) avaient une hypertension pulmonaire modérée avec une PAPS entre 35 et 50 mmHg, et 41% des patients (10 patients) avaient une PAPS>50 mmHg.

Enfin, l'étude de la fonction ventriculaire droite n'a pas retrouvé de corrélation statistiquement significative entre la fonction VD et le GMT (R:-0,002). Dans le groupe 1, la fonction VD était bonne chez 84% des patients (22 patients) et moyenne chez 15% des patients (4 patients). Dans le groupe 2, la fonction VD était bonne ou conservée chez 91% des patients et altérée chez 8% des patients (2 patients) (Tableau 3).

**Tableau 3:** Corrélation entre le gradient moyen transmitral et les paramètres cliniques, électrocardiographiques et écho cardiographiques étudiés

	Gradient moyen<10 mmHg (nombre de patients)	Gradient moyen>10 mmHg (nombre de patients)	Coefficient de corrélation de Pearson (R)
**Surface mitrale (RM serré ou très serré)**	26	24	-0,137
**Dyspnée**			0,586^+^
Stade II	21	7	
Stade III	4	10	
Stade IV	0	7	
**Fréquence cardiaque**			0,615^+^
<60 bpm	1	0	
Entre 60 et 100 bpm	25	11	
>100bpm	0	13	
**Pression artérielle systolique**			0,518^+^
<35 mmHg	17	3	
Entre 35 et 50 mmHg	6	11	
>50 mmHg	3	10	
**Fonction VD**			-0,002
Bonne	18	17	
Conservée	4	5	
Moyenne	4	0	
altérée	0	2	
**ECG**			0,038
Rythme régulier sinusal	14	12	
ACFA	12	12	

## Discussion

Avec la diminution de l´incidence du rhumatisme articulaire aigu dans les pays occidentaux, le rétrécissement mitral est devenu plus rare mais a également changé de présentation clinique, survenant chez des patients plus âgés avec une anatomie valvulaire plus altérée. Dans les pays en voie de développement, le rétrécissement mitral rhumatismal demeure une valvulopathie fréquente [1]. En effet, l'étiologie rhumatismale est la cause quasi exclusive du RM acquis de l'adulte. De rares cas des RM dégénératif liée à des calcifications massives de l'anneau mitral ont été rapportés. Des cas encore plus rares de RM dégénératif dus à une sténose aortique avec coulée calcaire atteignant la mitrale, à un lupus érythémateux systémique ou au syndrome des anti-phospholipides, à une cardiopathie carcinoïde ou à une fibrose endomyocardique ont été occasionnellement décrits [2]. Dans notre étude, l'étiologie rhumatismale est la cause du RM chez tous nos patients.

L'évaluation du RM est basée sur une démarche dans laquelle la définition du caractère serré du RM est une étape première et capitale avant de décider de la nécessité ou non du traitement du RM. L'évaluation de la sévérité du RM repose sur la mesure de la surface valvulaire mitrale (MVA). Plusieurs méthodes sont possibles et doivent souvent être réalisées conjointement. La planimétrie est la méthode de référence, mais doit être effectuée précisément au sommet de l'entonnoir mitral et nécessite un opérateur expérimenté. Le temps de demi-pression, ou PHT, est une méthode simple, mais pas toujours fiable en particulier chez les sujets âgés ou en cas de fibrillation auriculaire et elle n'est pas valide immédiatement après une commissurotomie mitrale percutanée. L'équation de continuité est relativement simple mais invalide en cas de fuite aortique ou mitrale associée et en cas de fibrillation auriculaire. La proximal isovelocity surface area est réputée complexe et nécessite une correction d'angle (mesure de l'angle formé par les feuillets valvulaire). Les nouvelles modalités d'imagerie, comme l'échographie tridimensionnelle ou le scanner, sont intéressantes et semblent moins operateurs-dépendantes [3]. La mesure de la surface valvulaire mitrale dans notre étude a été faite par méthode de planimétrie chez tous les patients. En effet, presque la moitié de nos patients (48%) étaient en ACFA, ce qui rend la méthode de mesure de la SM par PHT peu valide. Pour les valeurs seuils de **surface valvulaire mitrale** qui définissent un RM serré ou très serré, plusieurs discordances existent entre les différentes sociétés savantes. La société américaine d'échocardiographie (ASE) et l'association européenne d'échocardiographie (EAE) ont défini en 2009un RM « significatif » par une surface valvulaire mitrale (SVM) <1,5cm^2^ et un RM « sévère » par une SVM<1 cm^2^ [4].

La société européenne de cardiologie (ESC) a défini en 2012 un la sévérité du RM par une SVM <1cm² et un RM « significatif » à partir duquel une intervention par commissurotomie mitrale percutanée ou un remplacement valvulaire mitral peut être envisagée, par un SVM = à 1,5cm^2^ [5]. Quant à la société américaine de cardiologie (ACC/AHA), elle considéré dans ses guidelines pour la prise en charge des cardiopathies valvulaires en 2014, un RM sévère à partir d'une SVM = à 1,5cm^2^ et un RM très serré par une SVM ou= à 1cm^2^ [1]. La mesure des **gradients transmitraux** est une mesure simple qui utilise de doppler continu et la moins sujette à des erreurs; puisque les gradients sont dérivés de la mesure directe de la vélocité du flux transmitral. En coupe apicale des 4 cavités, ou parfois en coupe apicale 2ou3 cavités, le repérage du flux transmitral se fait aisément au doppler couleur ; il est parfois très excentré lorsque l'orifice est très remanié et il est alors utile de pouvoir aligner le faisceau doppler sur la zone du jet central, au maximum d'aliasing, là où le flux est laminaire [6]. On préfère le doppler continu par rapport au doppler pulsé, afin de ne pas écrêter les spectres, surtout si les flux sont très rapides. On fait ensuite le contournage de l'enveloppe spectrale, que toutes les machines d'échographie actuelles permettent de tracer directement sur l'écran et l'on détermine ainsi les gradients maximal et moyen à partir des vélocités maximales et moyenne, selon la formule de Bernoulli simplifiée. Le gradient moyen transmitral est beaucoup plus intéressant à considérer ; sa valeur n'excède pas normalement 3 mmHg. Mais en réalité, le GMT ne reflète que très imparfaitement la sévérité du RM [7, 8]. L'ASE 2009 a considéré la valeur du GMT comme élément d'appui ou d'orientation vers un RM serré si ce gradient est >10 mmHg sans l'inclure dans la définition du RM serré [4]. L'ACC/AHA 2014 a corrélé le RM serré à un GMT entre 5 et 10 mmHg sans inclure le GMT dans la définition de la sévérité du RM [1].

L'ESC 2012 a défini la sévérité du RM par une SVM<1 cm^2^ et un GMT>10 mmHg, à condition d'interpréter ce gradient en fonction de la fréquence cardiaque et chez un patient en rythme sinusal [5]. Dans notre série, 52% des patients avaient un GMT <10mmHg et 42% parmi eux avaient un RM très serré, ce qui suggère l'absence de corrélation directe entre une SVM <1 cm^2^ ou <1,5 cm^2^ et un GMT>10 mmHg, et que d'authentiques RM même très serré peuvent exister avec des GMT<10 mmHg. Le point commun entre les différentes sociétés savantes est la grande variabilité du GMT en fonction de plusieurs paramètres hémodynamiques, en particulier la régularité du rythme, la fréquence cardiaque, le débit cardiaque et l'existence d'une insuffisance mitrale associée [1, 4, 5, 9].

A noter que notre étude porte sur le RM serré pur sans valvulopathie significative associée et que tous nos patients avaient une fraction d'éjection ventriculaire gauche normale ou conservée, et donc la corrélation du GMT avec le débit cardiaque et l'IM n'ont pas été étudiés. La fréquence cardiaque est l'un des principaux facteurs qui influencent le GMT car ce dernier augmente en cas de tachycardie. En effet, la fréquence cardiaque au moment du calcul du GMT doit toujours être rapportée sur le compte rendu d'échocardiographie [4].

La régularité du rythme est un autre paramètre déterminant du GMT car ce dernier augmente en cas de tachy-ACFA et lors d'un cycle cours de fibrillation atriale. Il est communément admis qu'en cas de fibrillation atriale, il est indispensable de moyenner le résultat de 5 à 10 cycles avec le moins de variations de l'espace R-R possible et de réévaluer le patient après ralentissement de la fréquence cardiaque [9]. Ceci a conduit quelque équipe à considérer le « gradient moyen corrigé » qui représente la valeur du gradient moyen transmitral par rapport à la fréquence cardiaque [10]. L'analyse de la corrélation entre la régularité du rythme et le GMT dans notre étude n'a pas été statistiquement significative. En effet, 53% des patients qui avaient un GMT<10 mmHg étaient en Rythme régulier sinusal (RRS) et 46% (12 patients) étaient en fibrillation atriale. Pour les patients qui avaient un GMT>10 mmHg, la moitié des patients était en RRS et la moitié en fibrillation atriale. Ceci peut être expliqué par le fait que seulement le tiers de nos patients qui étaient en fibrillation atriale avaient une FC >100 bpm, contre 62% en fibrillation atriale avec une FC entre 60 et 100 bpm. Cependant, le GMT est plutôt un indicateur de tolérance hémodynamique du RM. En effet, dans notre série, nous avons retrouvé une corrélation significative entre le GMT et la sévérité de la dyspnée, l'existence d'une décompensation cardiaque et l'hypertension artérielle pulmonaire.

## Conclusion

Le gradient moyen transmitral est un bon indicateur de la tolérance du rétrécissement mitral, mais il reflète imparfaitement sa sévérité vue qu'il dépend de plusieurs paramètres hémodynamiques. D'authentiques rétrécissements mitraux très serrés peuvent exister avec des gradients moyens transmitraux <10 mm Hg, c'est pour cette raison que la valeur du GMT ne doit jamais être interprétée isolément.

### Etat des connaissances actuelles sur le sujet

- Les sociétés savantes internationales définissent le RM serré par une surface valvulaire mitrale = à 1,5 cm² et un RM très serré par une surface valvulaire mitrale <1cm2 avec un gradient moyen transmitral >10 mmHg;
- Pour d'authentiques rétrécissements mitraux serrés, on retrouve souvent un gradient transmitral discordant inférieur à 10 mmHg;
- Aucune étude n'a pu établir une corrélation entre le gradient transmitral et la tolérance fonctionnelle des rétrécissements mitraux serrés.

### Contribution de notre étude à la connaissance

- Absence de corrélation directe entre la sévérité du rétrécissement mitral et le gradient transmitral vue la présence d'authentiques rétrécissements mitraux serrés ou très serrés avec des gradients transmitraux inférieurs à 10 mm Hg;
- L'existence d'une corrélation significative entre le gradient transmitral et la tolérance fonctionnelle du rétrécissement mitral, en se basant sur la sévérité de la dyspnée et le nombre de décompensations cardiaques gauches;
- L'étude de la pression artérielle pulmonaire systolique dans les deux groupes de l'étude a révélé l'existence dans notre série d'une corrélation statistiquement significative entre la PAPS et le GMT.
